# Outbreak of Anthrax Associated with Handling and Eating Meat from a Cow, Uganda, 2018

**DOI:** 10.3201/eid2612.191373

**Published:** 2020-12

**Authors:** Esther Kisaakye, Alex Riolexus Ario, Kenneth Bainomugisha, Caitlin M. Cossaboom, David Lowe, Lilian Bulage, Daniel Kadobera, Musa Sekamatte, Bernard Lubwama, Dan Tumusiime, Patrick Tusiime, Robert Downing, Joshua Buule, Julius Lutwama, Johanna S. Salzer, Eduard Matkovic, Jana Ritter, Joy Gary, Bao-Ping Zhu

**Affiliations:** Uganda Public Health Fellowship Program, Kampala, Uganda (E. Kisaakye, A. Riolexus Ario, K. Bainomugisha, L. Bulage, D. Kadobera);; Ministry of Health, Kampala (A. Riolexus Ario, M. Sekamatte, B. Lubwama, P. Tusiime);; US Centers for Disease Control and Prevention, Atlanta, Georgia, USA (C.M. Cossaboom, D. Lowe, J.S. Salzer, E. Matkovic, J. Ritter, J. Gary, B. Zhu);; Ministry of Agriculture, Entebbe, Uganda (D. Tumusiime);; Uganda Virus Research Institute, Entebbe, Uganda (R. Downing, J. Buule, J. Lutwama);; US Centers for Disease Control and Prevention, Kampala (B.-P. Zhu)

**Keywords:** anthrax, *Bacillus anthracis*, bacteria, cutaneous anthrax, disease outbreaks, enteric infections, food-borne diseases, gastrointestinal anthrax, global health security, One Health, Uganda, zoonoses

## Abstract

On April 20, 2018, the Kween District Health Office in Kween District, Uganda reported 7 suspected cases of human anthrax. A team from the Uganda Ministry of Health and partners investigated and identified 49 cases, 3 confirmed and 46 suspected; no deaths were reported. Multiple exposures from handling the carcass of a cow that had died suddenly were significantly associated with cutaneous anthrax, whereas eating meat from that cow was associated with gastrointestinal anthrax. Eating undercooked meat was significantly associated with gastrointestinal anthrax, but boiling the meat for >60 minutes was protective. We recommended providing postexposure antimicrobial prophylaxis for all exposed persons, vaccinating healthy livestock in the area, educating farmers to safely dispose of animal carcasses, and avoiding handling or eating meat from livestock that died of unknown causes.

Anthrax is an acute zoonotic bacterial infection caused by *Bacillus anthracis*, a gram-positive, spore-forming bacteria that is thought to survive for as long as decades in the carcasses and burial sites of infected animals (*1*). Anthrax is transmitted to humans through handling or eating meat from infected animal carcasses, contact with their products (e.g., hair, wool, hides, bones), or by breathing in spores (*1*,*2*). 

Human anthrax infection is classified into 4 forms, depending on the route of exposure, each with a different incubation period: cutaneous (1–12 days), inhalational (1–60 days), gastrointestinal (1–6 days), and injectional (1–10 days) (*3*). Cutaneous anthrax is the most frequently reported form of human anthrax infection, accounting for up to 95% of cases (4). Both cutaneous and gastrointestinal anthrax outbreaks have been associated with handling or butchering infected animals and consuming their meat (5). It is estimated that each year 2,000–20,000 human anthrax cases occur worldwide (6). Most reported anthrax outbreaks occur in endemic areas in sub-Saharan Africa and Asia (1). 

On April 20, 2018, the Kween District of Uganda reported to the Ministry of Health 7 suspected cases of cutaneous anthrax from 2 neighboring villages, Kaplobotwo and Rikwo. We investigated to verify the existence of an anthrax outbreak, determine its scope, identify possible exposures, and recommend evidence-based control and prevention measures. 

## Methods

### Study Area

**Figure 1:**
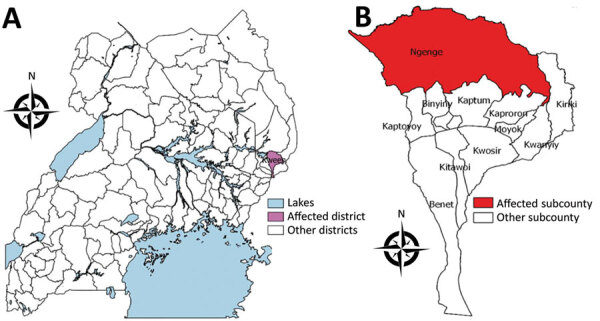
Area where anthrax outbreak occurred in April 2018, Kween District, Uganda. A) Location of Kween District in Uganda; B) Kween District, showing outbreak area.

Kween District is located in eastern Uganda (Figure 1). It is one of the so-called “cattle-keeping corridor” districts, where cattle-rearing is a major agriculture activity. 

### Case Definition

For this study, we defined a suspected cutaneous anthrax case as onset of skin vesicle or eschar, ≥2 cutaneous signs and symptoms (e.g., itching, redness, swelling), or any cutaneous sign or symptom plus regional lymphadenopathy, that occurred in a resident of Kaplobotwo and Rikwo during April 11–25, 2018. A suspected gastrointestinal anthrax case was defined as the acute onset of ≥2 signs or symptoms: abdominal pain, vomiting, diarrhea, or sore throat. A confirmed case was a suspected case followed up with a clinical specimen (blood or swab from skin lesion or vesicle) that tested positive for *B. anthracis* by real-time PCR (rPCR).

### Case Identification 

To identify anthrax cases and possible fatalities, we reviewed medical records from the 3 health facilities nearest the affected communities, Ngenge Health Center III and 2 private clinics. We also conducted a house-to-house search for cases in all 57 households of the 2 affected villages with the help of village leaders and members of the village health team. We developed a list of patients with details about age, sex, residence, date of onset of any signs or symptoms, treatment provided, specimens collected, results of laboratory tests conducted, and date of discharge if the patient was hospitalized. 

### Descriptive Epidemiology and Hypothesis Generation

We determined the epidemiology of the outbreak by date of symptom onset, location, and demographic characteristics of patients. To identify potential exposures leading to illness, we interviewed 12 suspected anthrax case-patients using convenience sampling in Kaplobotwo. We also conducted key informant interviews with village leaders. From interviews we learned of the sudden death of a cow owned by a resident of Kaplobotwo; the cow was subsequently butchered and eaten by some villagers. 

### Retrospective Cohort Study 

We conducted a retrospective cohort study in the more-affected village, Kaplobotwo, where 96% of the cases occurred, focusing on exposures, which we identified using the methods described. Using a standardized questionnaire developed by the team, we interviewed villagers present in the area at the time of the outbreak. We evaluated the association between exposure to the dead cow and illness onset separately by form of anthrax illness—cutaneous, gastrointestinal, or both. We computed the attack rate (AR) and risk ratio (RR) for each activity that resulted in exposure (e.g., butchering the cow, eating the meat) to assess the association between each individual exposure and subsequent illness. Using modified Poisson regression, we also evaluated the total number of cutaneous exposures for each person interviewed relative to the risk of cutaneous anthrax to assess the dose-response relationship (7). 

### Laboratory Investigations 

We collected 6 skin lesion swabs from patients with cutaneous-form anthrax and 8 blood specimens from patients with gastroenteritis-form anthrax and shipped the samples to the Uganda Virus Research Institute (UVRI; Entebbe, Uganda) for testing. The skin lesion swabs and blood specimens were tested at UVRI using rPCR following standard protocol (8). 

In addition, upon revisiting the village 1 month later, we tested a specimen collected from the dried hide of the dead cow using the Active Anthrax Detect test (AAD; InBios, https://inbios.com). AAD rapid test, a novel lateral-flow rapid diagnostic test that detects the capsular polypeptide of *B. anthracis*, was developed as a point-of-care test for presumptive human inhalation of anthrax spores and is available as an investigational use only– or research use only–product (9*,*10). We suspended the sample in 600 µL of sterile phosphate buffered saline, vortexed for 10 s, and, after pipetting the solution multiple times, applied 10 µL to the AAD cassette. 

We shipped a specimen from the same dried hide to the US Centers for Disease Control and Prevention (CDC; Atlanta, GA, USA) for confirmatory testing. DNA extraction on the specimen was performed using a QIAGEN Blood Mini Kit (QIAGEN, https://www.qiagen.com), and the resulting DNA was tested using real-time reverse transcription PCR for *B. anthracis* from the Laboratory Reference Network (https://emergency.cdc.gov/lrn) (11). A formalin-fixed sample from the dried hide was routinely processed, embedded in paraffin, and stained with hematoxylin and eosin, Lillie-Twort gram stain, and Warthin-Starry silver stain. Immunohistochemistry assays using mouse monoclonal antibodies targeting the *B. anthracis* cell wall and capsule were performed by using an immunoalkaline phosphatase polymer system as previously described (10*,*12).

### Trace-Forward Investigations and Environmental Assessment

We conducted in-depth interviews of the district health officer, the village leader, and the owner of the dead cow, as well as a convenience sample of 15 villagers who participated in the processing of the dead cow. The interviews were conducted to investigate the circumstances surrounding the death of the cow, identify people who participated in the butchering, and determine where the meat was distributed and how many people had received the meat. We also walked through the entire village to evaluate evidence of any other dead or sick livestock in the area. 

### Ethics Considerations

The Office of the Director General of Health Services, Ministry of Health of Uganda, gave the directive and approval to investigate this outbreak. The Office of the Associate Director for Science, Center for Global Health, US CDC, determined that this activity was in response to a public health emergency and not human subjects research. We obtained verbal informed consent from respondents ≥18 years of age or from their parents or guardians if respondents were <18 years of age. We stored all completed questionnaires in a secure location and stored the electronic data in a password-protected laptop to avoid disclosure of respondents’ personal information. Data were not shared outside of the investigation team and when being shared within the team, all personal identifying information was deleted in advance. 

## Results 

### Descriptive Epidemiology and Hypothesis Generation

We identified 49 cases of human anthrax, 46 suspected and 3 confirmed by rPCR testing. No human deaths were reported. The mean age of the 49 patients was 30 (range 1–84) years. Of the 49 cases, 13 (27%) had cutaneous anthrax only, 16 (33%) had gastrointestinal anthrax only, and 20 (41%) had both cutaneous and gastrointestinal anthrax. Among the 20 patients with both cutaneous and gastrointestinal anthrax, 3 had photophobia, and 2 of those 3 also had neck pain or stiffness, suggesting possible meningeal involvement (13) (Table 1). 

**Table 1:** Clinical manifestations of anthrax by form in patients during an outbreak, Kween District, Uganda, April 2018

Signs and symptoms	No. (%) patients			
	All cases, N = 49	Cutaneous-only, n = 13	Gastrointestinal-only, n = 16	Both, n = 20
Cutaneous				
Skin itching (pruritis)	35 (65)	12 (92)	0	20 (100)
Skin reddening (erythema)	25 (51)	12 (92)	0	13 (65)
Skin swelling (edema)	26 (53)	11 (85)	0	15 (75)
Skin vesicles	17 (35)	8 (62)	0	9 (45)
Skin eschar	9 (18)	3 (23)	0	6 (30)
Regional lymphadenopathy	15 (31)	4 (31)	0	11 (55)
Gastrointestinal				
Abdominal pain	37 (76)	2 (15)	16 (100)	19 (95)
Diarrhea	28 (57)	0	12 (75)	16 (80)
Bloody diarrhea	9 (18)	0	6 (38)	3 (15)
Sore throat	13 (27)	0	6 (38)	7 (35)
Vomiting	10 (20)	0	3 (19)	7 (35)
Swollen neck lymph nodes	2 (4.1)	0	1 (6.3)	1 (5.0)
Systemic				
Fever	25 (51)	2 (15)	8 (50)	15 (75)
Lethargy	24 (49)	1 (7.7)	9 (56)	14 (70)
Anorexia	13 (27)	0	6 (38)	7 (35)
Difficulty breathing	5 (10)	0	1 (6.3)	4 (20)
Cough	5 (10)	0	1 (6.3)	4 (20)
Headache	3 (6.1)	0	0	3 (15)
Other				
Photophobia	3 (6.1)	0	0	3 (15)
Neck pain or stiffness	2 (4.1)	0	0	2 (10)

Key informant interviews indicated that a cow had died suddenly on April 11, 2018, at the residence of a Kaplobotwo resident. Subsequently, the dead cow was skinned and butchered. Most of the adults in Kaplobotwo participated in the butchering and handling of the meat, and many villagers took the meat home to eat. Some of the meat was also sold to neighboring Rikwo. According to local leaders, Kaplobotwo had a total of 234 residents and Rikwo a total of 120 residents. When we analyzed the geographic locations of the cases, 47 (96%) occurred in Kaplobotwo (AR 20%, 47/234) and 2 (4.1%) occurred in Rikwo (AR 1.7%, 2/120). In Kaplobotwo, all cases were within a 600-meter radius of the site where the dead cow was skinned and butchered. 

**Figure 2:**
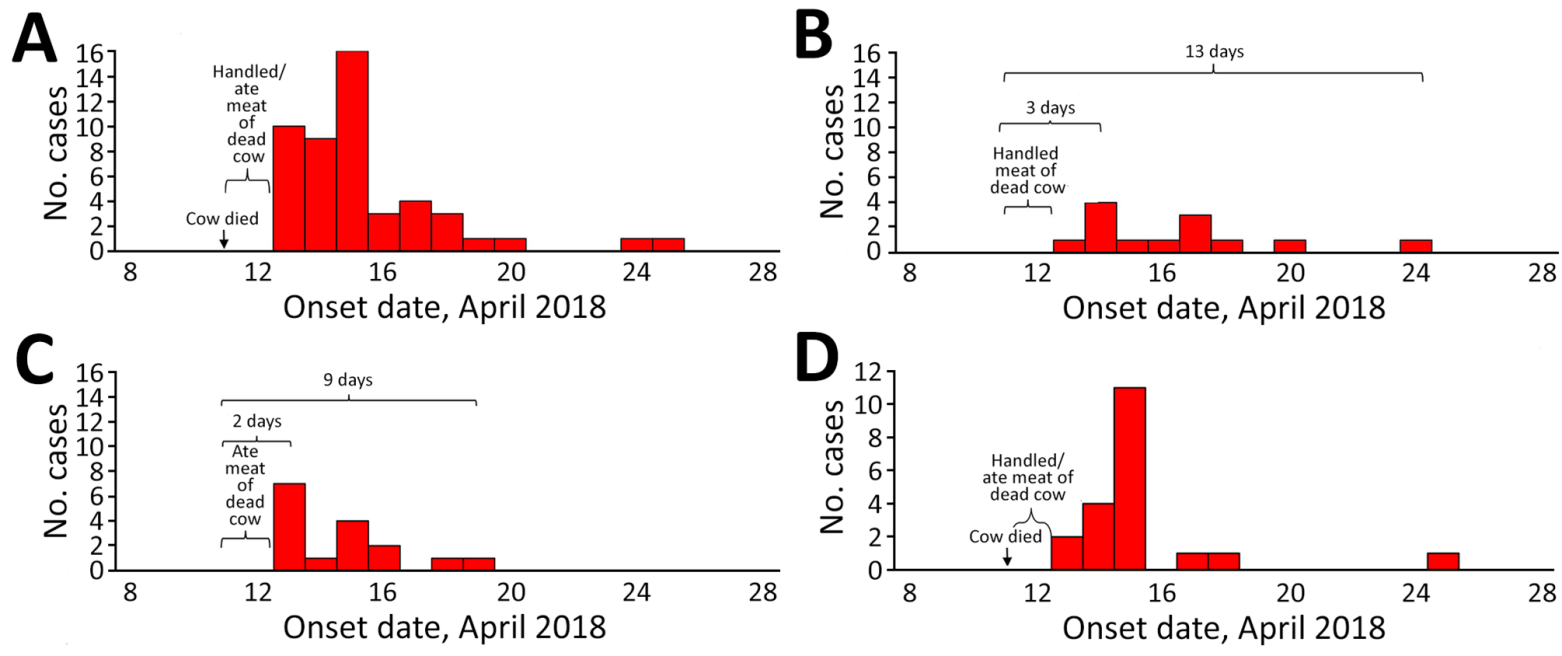
Distribution by date of onset of cases in anthrax outbreak that occurred in April 2018, Kween District, Uganda. A) All anthrax cases; B) cutaneous-only cases; C) gastrointestinal-only cases; D) cases of both cutaneous and gastrointestinal anthrax.

The epidemic curve showed that, after the death and processing of the cow, cases began to appear on April 13, and the number of cases rose, peaking on April 15, suggesting a point-source outbreak. After that, the onset of cases declined, the last being on April 25 (Figure 2, panel A). When the epidemic curve was stratified by anthrax forms (Figure 2, panels B–D), the intervals from exposure to the peak of the epidemic curve was 3 days for cutaneous and 2 days for gastrointestinal. The interval from the initial exposure (April 11–12, 2018) to the end of the epidemic curve was 12–13 days for onset of cutaneous anthrax and 8–9 days for onset of gastrointestinal anthrax. Of the 12 suspected case-patients who participated in the hypothesis-generation interview, 100% carried and ate the meat of the dead cow and were involved in the cutting or butchering, 50% participated in the cleaning of the waste site after the carcass was processed, and 33% participated in skinning the dead cow before butchering. 

### Retrospective Cohort Study Findings 

In our retrospective cohort study in Kaplobotwo, we interviewed 141 persons who resided in the village during April 2018 and therefore could have been exposed to anthrax. Among these 141 villagers, anthrax developed in 47 (AR 33%); cutaneous anthrax developed in 33 (AR 23%), and gastrointestinal anthrax developed in 34 (AR 24%). By anthrax form, the ARs were 9.2% for cutaneous-only, 9.9% for gastrointestinal-only, and 14% for combined cutaneous and gastrointestinal anthrax. 

Male residents had a lower AR than female residents for cutaneous-only anthrax (6.5% vs. 13%); however, this difference was not significant (p = 0.175 by Fisher exact test). Conversely, male residents had higher ARs than female residents for both the gastrointestinal-only form (16% vs. 3.1%; p = 0.012 by Fisher exact test) and the combined cutaneous and gastrointestinal form (19% vs. 7.8%; p = 0.039 by Fisher exact test). The cutaneous-only form affected adults more than children, whereas the gastrointestinal-only form affected young children (≤5 years) and older adults (>30 years) more than older children (5–17 years) and young adults (18–29 years). The mixed cutaneous and gastrointestinal form affected all age groups approximately equally (Table 2). 

**Table 2:** Anthrax attack rates by age and sex for each form, Kaplobotwo, Kween District, Uganda, April 2018*

Anthrax form	Total cohort	No. cases	AR, %
All anthrax	141	47	33
Sex			
M	77	32	42
F	64	15	23
Age range, y			
0–5	30	12	40
6–17	27	6	22
18–29	27	7	26
30–59	40	16	40
≥60	17	6	35
Cutaneous-only	141	13	9.2
Sex			
M	77	5	6.5
F	64	8	13
Age range, y			
0–5	30	1	3.3
6–17	27	1	3.7
18–29	27	3	11
30–59	40	6	15
≥60	17	2	12
Gastrointestinal-only	141	14	9.9
Sex			
M	77	12	16
F	64	2	3.1
Age range, y			
0–5	30	5	17
6–17	27	1	3.7
18–29	27	1	3.7
30–59	40	5	13
≥60	17	2	12
Cutaneous and gastrointestinal	141	20	14
Sex			
M	77	15	19
F	64	5	7.8
Age range, y			
0–5	30	6	20
6–17	27	4	15
18–29	27	3	11
30–59	40	5	13
≥60	17	2	12
*AR, attack rate.			

In the retrospective cohort study, certain activities were significantly associated with developing cutaneous anthrax: carrying the dead cow from the place of death to the place of butchering (RR 4.3, 95% CI 2.4–7.8), participating in the skinning of the cow (RR 4.2, 95% CI 2.6–6.7), participating in cutting and butchering of the dead cow (RR 4.9, 95% CI 3.2–7.9), participating in the removal of the organs (RR 3.5, 95% CI 2.1–6.0), carrying the skin of the dead cow from the butchering site to homes (RR 4.5, 95% CI 2.9–6.9), carrying the cut meat from the place of butchering to homes (RR 4.3, 95% CI 2.4–7.8), and cleaning the waste site after the butchering (RR 4.2, 95% CI 2.6–6.7). The number of cutaneous exposures for each person ranged from 0 to 7. Of the 141 persons who participated in the cohort study, 99 (70%) had no exposures at all, 6 (4.3%) reported only 1 exposure, 22 (16%) reported 2 exposures, 4 (2.8%) reported 3 exposures, 2 (1.4%) reported 4 exposures, 1 (0.71%) reported 5 exposures, 2 (1.4%) reported 6 exposures, and 5 (3.6%) reported having all 7 exposures. For each additional cutaneous exposure, the risk for cutaneous anthrax increased by 30% (RR 1.4, 95% CI 1.3–1.5). 

Eating meat from the dead cow was significantly associated with gastrointestinal anthrax (RR ¥, 95% CI 4.3–¥ by Fisher exact test; p = 0.00). Of the 95 persons who ate the cow meat, eating meat that was boiled for ≤30 minutes was significantly associated with gastrointestinal anthrax (RR 2.5, 95% CI 1.5–4.1); we found that boiling meat for >60 minutes was protective compared with the shorter cooking time (RR 0.34, 95% CI 0.18–0.67) (Table 3). 

**Table 3:** Retrospective cohort study on anthrax risk factors by form during outbreak, Kaplobotwo, Kween District, April 2018

Form	Cases			Attack rate, %		RR (95% CI)
	Exposed	Nonexposed		Exposed	Nonexposed	
Cutaneous anthrax						
Carried dead cow	37	104		54	13	4.3 (2.4–7.8)*
Participated in skinning	10	131		80	19	4.2 (2.6–6.7)*
Participated in cutting/butchering	10	131		90	18	4.9 (3.2–7.5)*
Participated in removing organs	10	131		70	20	3.5 (2.1–6.0)*
Carried the skin of the dead cow	8	133		88	20	4.5 (2.9–6.9)*
Carried cut meat	37	104		54	13	4.3 (2.4–7.8)*
Cleaned the waste	10	131		80	19	4.2 (2.6–6.7)*
For every additional exposure*						1.4 (1.3–1.5)*
Gastrointestinal anthrax						
Ate meat from dead cow, total	95	46		35	0	¥ (4.3–¥)*
Ate undercooked meat	9	86		78	31	2.5 (1.5–4.1)*
Cooking/boiling time						
Boiled meat >60 vs. ≤30min	41	22		22	64	0.34 (0.18–0.67)*
Boiled meat 31–60 vs. ≤30min	17	22		35	64	0.55 (0.27–1.1)
*Estimated using modified Poisson regression.						

### Laboratory Investigation Findings

Of the 6 skin lesion swabs collected, 3 tested positive for *B. anthracis* DNA by rPCR at UVRI. All 8 blood samples were negative for *B. anthracis* by rPCR at UVRI. It should be noted that, at the time of specimen collection, all patients had already started and some had completed antimicrobial treatment. A sample from the dried hide of the cow, taken 1 month after the initial visit to the village, tested positive by AAD in the field and was confirmed to be positive for *B. anthracis* by both rPCR and immunohistochemistry at CDC. 

### Trace-Forward and Environmental Investigation 

According to the village leader, after the cow died on April 11, 2018, a total of 10 residents of Kaplobotwo participated in butchering, skinning, and carrying meat from the cow, and most of the villagers ate meat from the dead cow. Environmental investigations found that the village was near the Panupe Game Reserve. Piles of animal bones were found in the livestock grazing fields, indicating past animal deaths. Interviews with community leaders revealed that these were remains from animals that had died suddenly and were abandoned in the grazing fields. 

Some of the meat from the dead cow was reportedly sold to 2 neighboring villages, Rikwo and Tukumo. Due to resource limitations, we were unable to conduct house-to-house searches for cases in these 2 villages; instead, we contacted the village leaders for case finding. In Rikwo, a family of 2 bought the meat from a meat broker, and gastrointestinal symptoms developed in both family members after they ate the meat. The owner of a bar in the same village also bought the meat, boiled it overnight, poured out the broth from the boiling pot the next morning, fried the boiled meat, and sold it to 28 patrons the next day. None of the patrons reported any gastrointestinal symptoms. In Tukumo, the meat was sold to a bar, a restaurant, and an unknown number of individual families. The village leader was aware of 23 persons who bought and ate the meat; however, he did not know of anyone who had reported gastrointestinal or cutaneous anthrax symptoms. 

## Discussion

On the basis of epidemiologic, laboratory, and environmental assessments, we determined that this was a point-source cutaneous and gastrointestinal human anthrax outbreak associated with handling and eating meat from a cow that had died from confirmed anthrax infection. Results from this investigation were consistent with those in other anthrax outbreak investigations in which anthrax patients were infected through contact with diseased livestock or contaminated animal products (14–17). 

In our study, although the cause of the cow’s death was unknown at the time of death, subsequent laboratory testing confirmed anthrax in the dried hide of the cow. In this area, when a cow is butchered, it is customary to share meat with all households in the village. In this case, this custom exposed the entire village to anthrax. Butchering anthrax-infected animals and disposing of carcasses and waste in environments where ruminants live and graze, combined with limited vaccination of livestock against anthrax, enables further environmental contamination with *B. anthracis* spores and propagation of anthrax outbreaks in animals and zoonotic transmission to humans (18). Findings from this investigation are consistent with findings from a previous study in Kuwirirana ward, Gokwe North, Zimbabwe, in which anthrax also resulted from contact with and consumption of anthrax-infected carcasses (19). 

Among people, anthrax infection is typically an occupational disease, most common among farmers and workers with occupational activities that involve handling animals and animal products, such as the herders, butchers, and others. Infections may also occur among persons who consume infected meat (4*,*20*,*21). In this outbreak, cutaneous-only anthrax affected adults more than children, probably because adults were more likely to have been engaged in handling and processing the dead cow. 

Spores of *B. anthracis* are refractory to inactivation by boiling and, in this outbreak, eating undercooked meat was significantly associated with developing gastrointestinal anthrax. Conversely, boiling meat for >60 minutes appeared to be protective among persons who ate it, possibly because that length of time could have allowed the heat to rise to a temperature sufficient to inactivate a portion of the spores. Whether or not this actually occurred is unclear. Findings in this study are consistent with those found in a study in Bangladesh in which high rates of cutaneous anthrax but few gastrointestinal anthrax cases occurred in a community that had cooked the meat longer (22). 

In addition, the risk for gastrointestinal anthrax remained high even when the meat was well cooked (AR 31%) or boiled for >60 minutes (AR 22%). According to World Health Organization guidelines, “any animal that is sick, behaves strangely or has died suddenly should not be used for food or for making any product, as it may have succumbed to an infectious disease” (23). Following these guidelines can safeguard both animal products and persons involved in handling them. 

This study had some limitations. In this outbreak, *B. anthracis* was confirmed by rPCR in 3 of the 6 skin-lesion swab specimens, as well as from the dried hide of the cow. However, the 8 blood specimens from patients with gastroenteritis were negative for *B. anthracis* by both rPCR and culture. These negative findings might be explained by the fact that all patients were already under antimicrobial treatment at the time of specimen collection. Whereas clinical and epidemiologic characteristics strongly suggested gastrointestinal anthrax, we were unable to provide definitive proof without laboratory confirmation. Clinical signs and symptoms of both cutaneous and gastrointestinal anthrax are nonspecific; therefore, some of the identified cases found might actually have been noncases. In addition, the dried hide of the implicated cow tested positive by AAD rapid test. There is great utility for a rapid diagnostic test for presumptive diagnosis of anthrax under field conditions, but care must be taken when interpreting the results of this test. Recent work has identified that the specificity of this assay decreases with carcass age (>24 hours after death), so parallel confirmatory testing is critical when interpreting results from this test (24). Also, trace-forward investigation indicated that some meat from the implicated cow might have been sold to neighboring villages, but no house-to-house search was conducted in those villages, possibly resulting in undercounting of cases. 

This investigation highlights an outbreak of human cutaneous and gastrointestinal anthrax among persons handling and eating meat from a cow that died of presumed anthrax. As a result of our findings, we made several recommendations to the communities: routinely vaccinate livestock; continue education and mobilization for anthrax; administer antimicrobials to all persons identified with anthrax and prophylaxis to exposed community members; use rapid diagnostic tests at the district level to quickly provide presumptive evidence of anthrax in animal carcasses; and safely bury carcasses under supervision. For burial, carcasses should be disinfected at the site of death with 12.5% formalin solution and buried in a pit >6 feet deep with the bottom of the pit ≥3 feet above the water table. We also recommended building capacity and the awareness of healthcare workers to obtain samples from patients before beginning drug administration. 

The investigation team worked with the district to conduct community health education on these recommendations and about the dangers of eating meat from animals found dead. We also provided antimicrobial treatment (ciprofloxacin and doxycycline) to all identified patients, offered postexposure antimicrobial prophylaxis to carcass-disposal team members and exposed community members, replenished antimicrobials at Ngenge Health Center III, and provided personal protective equipment and training in its use to the carcass disposal teams. Finally, we advocated for prompt reporting of suspected anthrax cases to the district health office, district veterinary office, and the national One Health coordinator. 
